# Impacts of autofluorescence on fluorescence based techniques to study microglia

**DOI:** 10.1186/s12868-022-00703-1

**Published:** 2022-03-31

**Authors:** Haozhe Zhang, Chen Tan, Xiaoyue Shi, Ji Xu

**Affiliations:** 1 Department of Pharmacology, School of Basic Medical Sciences, Zhengzhou University, Zhengzhou, Henan China; 2 Institute of Neuroscience, School of Basic Medical Sciences, Zhengzhou University, Zhengzhou, Henan China; 3 Translational Medicine Research Center, People’s Hospital Of Zhengzhou, Zhengzhou, Henan China

**Keywords:** Microglia, Autofluorescence, Flow cytometry, Immunofluorescence, Live imaging

## Abstract

**Background:**

Microglia, the resident immune cells in the central nervous system, accrue autofluorescent granules inside their cytoplasm throughout their lifespan. In this report, we studied the impacts of autofluorescence on widely used fluorescence-based techniques to study microglia, including flow cytometry, immunofluorescence staining, and live imaging.

**Results:**

The failed attempt of using fluorescein isothiocyanate (FITC) conjugated antibody to detect lymphocyte-activation gene 3 protein in microglia prompted us to compare the sensitivity of FITC, phycoerythrin (PE) and allophycocyanin (APC) conjugated antibodies to detect surface protein expression in microglia. We found that PE outperformed FITC and APC as the fluorophore conjugated to antibody for flow cytometry by overcoming the interference from microglia autofluorescence. To identify the location and source of microglia autofluorescence, we did confocal imaging and spectral analysis of microglia autofluorescence on fixed brain tissues, revealing that microglia autofluorescence emitted from cytoplasmic granules and displayed a multi-peak emission spectrum. We recommended removing autofluorescence by lipofuscin removing agents when staining intracellular proteins in microglia with the immunofluorescence techniques. On live brain slices, autofluorescent granules reduced the amplitudes of calcium signals in microglial somata derived from GCaMP6s fluorescence and thus needed to be excluded when selecting regions of interest (ROI).

**Conclusions:**

In conclusion, autofluorescence is a critical factor to consider when designing experiments and interpreting results based on fluorescence-based techniques to study microglia.

**Supplementary Information:**

The online version contains supplementary material available at 10.1186/s12868-022-00703-1.

## Background

Microglia are tissue-resident immune cells in the central nervous system. In physiological states, the branches and fine filopodia extend from somata and continuously survey the environment [1]. Once activated by pathological stimuli, microglia alter their functional states to either contribute to or mitigate disease progression. Activated microglia can secrete a spectrum of chemokines and cytokines, which could affect other cells’ function and survival in the central nervous system (CNS) [2, 3]. Microglia are also phagocytes, which engulf and digest the structures of damaged or stressed cells in the CNS [4]. By engaging these processes, microglia are an essential component of the development, homeostasis, and disease progression in the central nervous system.

Fluorescence-based techniques, including flow cytometry, immunofluorescence, and live imaging, have been extensively employed to study microglia. Since microglia can be isolated from a CNS single-cell suspension, flow cytometry has been extensively used to study protein expression in microglia. Flow cytometry analysis of CD11b and CD45 markers can identify two distinct populations. One is CD11b^+^CD45^low^ cells which represent parenchyma microglia derived from yolk sac progenitor cells. The other is CD11b^+^CD45^high^ cells which represent border associated macrophages or infiltrated macrophages derived from bone marrow monocytes. The surface expression of other molecules under investigation in these populations can be further analyzed by multicolor flow cytometry after staining with antibodies conjugated with variant fluorophores. Excitation and emission spectrum, as well as quantum yields, are the main characteristics to consider when choosing fluorophores. The immunofluorescence techniques have been used to study various aspects of microglia on fixed brain slices, including morphology, proliferation, phagocytosis, and etc., commonly by co-staining the molecule under investigation and a microglia-specific marker, such as IBA-1, TMEM119, and P2RY12 [5, 6]. Live imaging on brain slices and in vivo has been employed to study microglia morphology, surveillance, migration, and calcium signaling [7–10].

Lipofuscin-like autofluorescent granules have been reported to accumulate in microglia from aging rodents, although the exact substances emitting autofluorescence are still not clear [11–13]. Autofluorescent microglia may represent a subpopulation with unique photophysical, histological and functional properties which remains a constant percentage throughout the animals’ lifespan [14]. However, how the autofluorescence would affect the fluorescence-based techniques to study microglia has not been reported.

In this report, we studied how autofluorescence would affect the application of flow cytometry, immunofluorescence, and live fluorescence imaging of microglia. We found that the application of phycoerythrin (PE) conjugated antibodies allowed flow cytometry to have higher sensitivity than fluorescein isothiocyanate (FITC) and allophycocyanin (APC) conjugated antibodies to detect protein expression in microglia, by overcoming the interference from microglia autofluorescence. Imaging of microglia on fixed and live tissues revealed that cytoplasmic granules in microglia CD68 positive lysosomes emitted autofluorescence. These autofluorescent granules from adolescent mice can potentially confound the interpretation of immunofluorescence results and reduce the sensitivity of detecting calcium signals in microglial somata.

## Methods

### Animals

All mouse experiments were approved by the Animal Care and Use Committee at Zhengzhou University. C57BL/6 mice, *Cx3cr1*^GFP^ mice (JAX # 005582), and Ai96(RCL-GCaMP6s) (JAX # 028866) mice were obtained from Jackson Labs, Ltd. *Cx3cr1*^CreER^ mice were kindly provided by Dr. Steffen Jung from Weizmann Institute of Science. *Cx3cr1*^GFP^ and *Cx3Cr1*^CreER^ mice were maintained as heterozygous mice. Ai96 (RCL-GCaMP6s) were maintained as homozygous. *Cx3Cr1*^CreER^: *RCL-GCaMP6s* were obtained by crossing *Cx3Cr1*^CreER^ heterozygous mice with Ai96 (RCL-GCaMP6s) homozygous mice and screening *Cx3Cr1*^CreER^ heterozygous offsprings by PCR genotyping. Tamoxifen (75 mg/kg) was given intraperitoneally for 5 consecutive days at the age of 2 months to activate CreERT2 recombinase, which can excise the stop cassette in the genomic DNA of C *Cx3Cr1*^CreER^: *RCL-GCaMP6s* mice and thus allow the transcription of downstream GCaMP6s. LPS from Escherichia coli O127:B8 (Sigma, L5024) was injected intraperitoneally (5 µg/g body weight) and brain tissue was collected 16 h after injection. Both males and female were used and balanced numbers of sexes were mixed in our analysis and reports.

### Primary microglia isolation

Primary mouse microglia were isolated as previously described with slight modification [15]. In brief, 7 to 9 weeks old mice were euthanized and transcardially perfused with ice-cold 0.0356% heparin sodium solution. The brain was removed and placed in an EP tube containing ice-cold 1X PBS. The whole brain was cut into pieces with an ophthalmic scissor, passed through a 22 gauge needle 5 times, and filtered with 70-μm filters. Brain homogenate was applied to a percoll gradient, and after a 30 min spin at 500 g, cells were collected from the 30–70% interphase, pelleted, and washed.

### Flow cytometry

Several strategies were used to block the suspected binding of antibodies to Fcγ, including CD16/CD32 antibody (clone 93, Biolegend), CD16.2 antibody (clone 9E9, Biolegend), and mouse IgG (Solarbio). For antibody labeling, cells in the 200 µL ice-cold 1 × PBS were stained with CD11b (clone M1/70, BD Biosciences), CD45 (clone30-F11, Biolegend) and LAG-3 (clone C9B7W, Biolegend) or TIM-3 antibodies (clone RMT3-23, Biolegend) for 30 min on ice. Flow cytometry analysis was performed on a BD FACScanto. Data were analyzed using FlowJo software (TreeStar).

### Confocal imaging on fixed brain slices

Male and female mice were anesthetized with pentobarbital (100 mg/kg, i.p.) and transcardially perfused with PBS followed by 4% paraformaldehyde (PFA) in PBS, pH 7.4. Brains were post-fixed overnight in 4% PFA buffer, followed by cryoprotection in 30% sucrose in PBS for at least 48 h. Mouse brains were then embedded in Neg-50 frozen section medium (Fisher Scientific), sectioned using a cryostat at 40 µm, and mounted on coverslips for confocal imaging.

Immunostaining was performed as previously described [16]. For pretreatment of slices by lipofuscin quencher, slices were dipped in 1X TrueBlack^®^ in 70% ethanol for 30 s before primary antibody incubation. Sections were then incubated in primary antibodies (Iba-1, #019-19741, Wako; CD68, clone #FA-11, Biolegend) overnight before secondary antibody incubation and mounting.

Confocal fluorescence images were taken using Plan Apo 60 × 1.4 NA oil-immersion objective lens and the Nikon A1 confocal laser-scanning microscope. We used the 488 nm laser to excite GFP, with the intensity adjusted to 1% of the maximum output. The emitted light pathway consisted of an emission band pass filter (500–550 nm) before the photomultiplier tube. Autofluorescence was excited by the 561 nm laser line at 1% of the maximum output. The emitted light pathway consisted of a 570–620 nm emission filter. The spectral images were taken by an A1-DUVB-2 GaAsP detector unit (400–720 nm, 10 nm per step).

### Brain slice preparation

For brain slice preparation, mice were deeply anesthetized with isoflurane and decapitated. Coronal brain slices (300 μm thickness) were prepared in chilled cutting solution comprising the following (in mM): 110 NaCl, 2.5 KCl, 1.25 NaH_2_PO_4_, 2 CaCl_2_, 7 MgCl_2_, 25 d-glucose, 75 sucrose bubbled with 95% O_2_/5% CO_2_. During incubation, the slices were submerged at room temperature in aCSF comprising the following (in mM): 126 NaCl, 25 NaHCO_3_, 10 d-glucose, 2.5 KCl, 1.3 MgCl_2_, 2.4 CaCl_2_, and 1.24 NaH_2_PO_4_, bubbled with 95% O_2_/5% CO_2_. Brain slices were incubated at room temperature throughout the day. Slices were transferred to a recording chamber perfused with aCSF at a rate of 1–2 ml/min at 33 °C.

### Cranial window surgery

7 to 9 weeks old mice were implanted with a chronic cranial window. Briefly, mice were shaved head hair and injected with carprofen (20 mg/kg). During surgery, mice were anesthetized with isoflurane (5% for induction; 1–2% for maintenance) and placed on a heating pad. Using a dental drill, a circular craniotomy of > 3 mm diameter; the craniotomy center was around the limb/trunk region of the somatosensory cortex. A 70% ethanol-sterilized 3 mm glass coverslip was placed inside the craniotomy. A dental Resin cement (3M-U200) was applied and air dried. A dental resin cement was applied to the rest of the skull, except for the region with the window. The dental glue was used to attach a custom-made head plate onto the dental resin cement of the skull. Mice were allowed to recover from the cranial window surgery for 2–4 weeks before the commencement of chronic imaging. Only surviving mice with a clear glass window were used for the imaging studies.

### 2-photon imaging on live brain slices and in vivo

2-photon fluorescence images were taken using the Nikon NIR Apo 40 × 0.8NA water immersion objective lens and the Nikon A1 multi-photon microscope. On live brain slices, we used lock phased Coherent Chaemeleon 2-photon laser at 920 nm to excite GCaMP6s, with the intensity adjusted to 10% of the maximum output. The emitted light pathway consisted of an emission bandpass filter (505–525 nm) before the IR NDD. The spectral images were taken by an A1-DUVB-2 GaAsP detector unit (400–720 nm, 10 nm per step). In vivo, 820 nm laser was used to excite GFP and autofluorescence in microglia from *Cx3cr1*^GFP^ mice. The emission light of GFP was passed through a 500–550 nm band pass filter and autofluorescence through a 600–656 nm band pass filter.

### Quantification and statistical analysis

Sample sizes were based on similar previously published work. The results of statistical comparisons, n numbers and p values are shown in the figure panels or figure legends with the average data. All statistical tests were run in GraphPad Prism 8. The graphs were created in GraphPad Prism 8 or Origin 8 and assembled in Powerpoint 2016. No data has been excluded from the analysis. Since the mice used in our study are all in congenic C57BL/6 J background and thus no cofounder would be expected to affect the comparison between different treatment groups, therefore, animal selection has not been randomized. Since no subjective methods have been used in our study and thus no bias during data collection would be expected, investigators were not blind to the groups during data collection. The normality of the data distribution was determined using the Shapiro–Wilk test before appropriate statistical methods were chosen. If the data were normally distributed, two tailed Student’s *t* test or ANOVA were used. If data were not normally distributed, non-parametric Mann–Whitney test was used.

## Results

### FITC conjugated antibody fails to detect LAG-3 expression in microglia

**Fig. 1:**
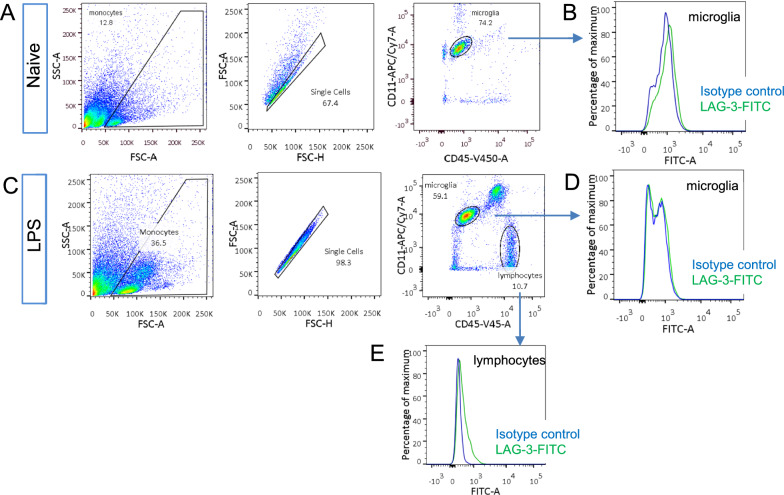
Gating strategies and detection of LAG-3 expression by FITC conjugated LAG-3 antibody.** A** Gating strategy of isolating single CD11^+^/CD45^low^ cells from the brains of PBS injected mice. Single-cell suspension from the brains of naïve mice involved pre-gating by forward scatter area (FSC-A) vs. side scatter area (SSC-A) plot, and forward scatter height (FSC-H) vs. FSC-A density plot to select singlets. Microglia were then identified by the high expression of CD11b and the low expression of CD45. **B** Distribution of fluorescence intensities from FITC conjugated LAG-3 antibody and isotype control antibody treated microglia. **C** Gating strategy of isolating single CD11^+^/CD45^low^ microglia cells and CD11^−^/CD45^high^ lymphocytes from the brains of LPS injected mice. **D**, **E** Distribution of fluorescence intensities from FITC conjugated LAG-3 and isotype control antibody-treated microglia (**D**) and lymphocytes (**E**) from LPS injected mice. All data were replicated in four independent experiments

We first studied how autofluorescence would affect flow cytometry by studying lymphocyte-activation gene 3 (LAG-3) expression in microglia cells. The available RNA-seq data and our RT-PCR results have strongly suggested the expression of LAG-3 mRNA in them (Additional file 1: Fig. S1A) [17]. Since flow cytometry has been extensively used to study LAG-3 expression and function in T cells, we prepared single-cell suspension from adult mice cortex and used flow cytometry to detect LAG-3 protein expression in microglia. FITC conjugated LAG-3 antibody was used for our purpose. Microglia cells were gated as CD11^+^/CD45^low^ cells from the brains of PBS injected 7–9 weeks old mice (Fig. 1A, B). Then, FITC signals from isotype control antibody and LAG-3 antibody-treated microglia were compared. From naive mice, FITC conjugated LAG-3 antibody-treated microglia presented a negligible LAG-3 specific signal comparing to isotype control antibody-treated ones (Fig. 1C). Since LPS induced inflammation can increase the expression of certain inflammation-related genes, we also performed flow cytometry on immune cells from the brains of mice injected with LPS intraperitoneally. Microglia still didn’t show a LAG-3 specific signal (Fig. 1D). However, infiltrated lymphocytes from the cortex of these mice, gated as CD11^−^/CD45^+^ cells, presented a LAG-3 specific signal (Fig. 1E). Therefore, FITC conjugated LAG-3 antibody failed to detect LAG-3 protein in microglia from adult mice.

### PE-conjugated antibody outperforms APC and FITC conjugated antibodies in detecting surface molecules in microglia

**Fig. 2:**
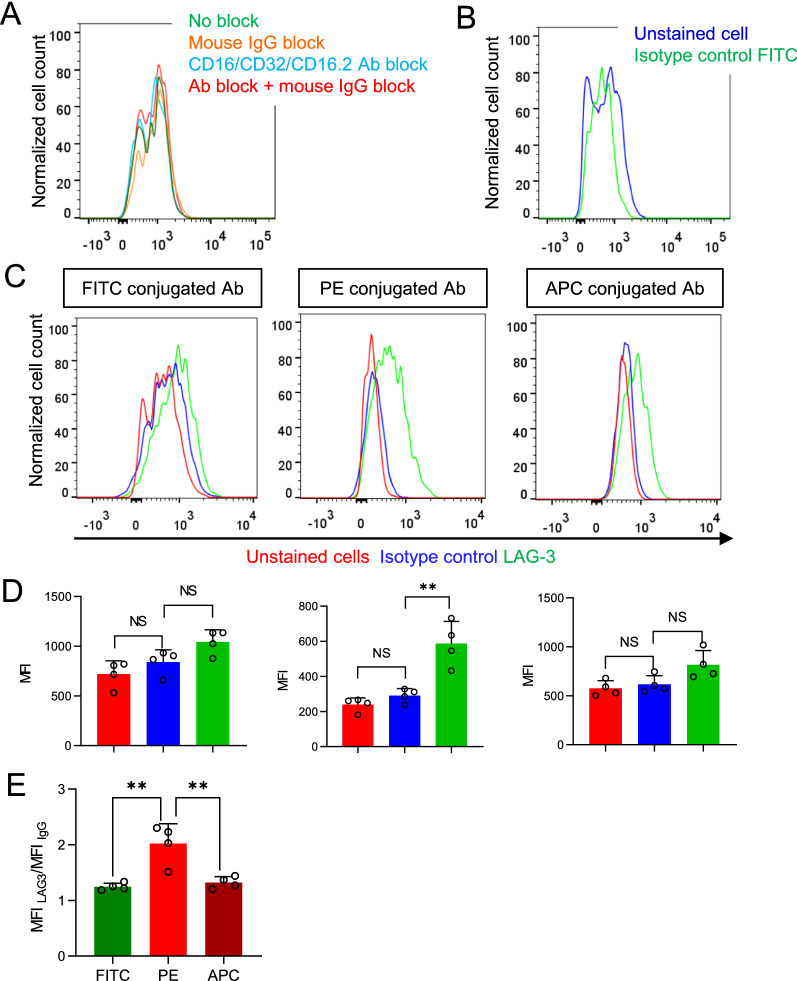
PE-conjugated antibody outperforms FITC and APC conjugated antibodies in detecting microglia LAG3.** A** Distribution of fluorescence intensities from FITC conjugated isotype control antibody labeled microglia with the previous blocking by mouse IgG, CD16/CD32/CD16.2 antibodies, or the combination of mouse IgG and CD16/CD32/CD16.2 antibodies. **B** Distribution of fluorescence intensities from nontreated microglia and isotype control antibody treated microglia. **C** Distribution of fluorescence intensities from nontreated microglia and FITC, PE, and APC conjugated LAG-3 antibodies and their respective isotype control antibodies treated microglia. **D** Median fluorescence intensity (MFI) of microglia as treated in (**C**). **E** Comparison of MFI _LAG-3_/MFI _IgG_ of FITC, PE, and APC conjugated LAG-3 antibodies (n = 4 mice). Bars represent the means ± standard deviation (SD). Comparisons were made by one-way ANOVA test with Tukey’s post hoc multiple comparisons test. *P < 0.05; **P < 0.01; NS, not significantly different

We speculated that the non-specific binding of antibodies to microglia may cause a high fluorescence background and lower the sensitivity of flow cytometry to detect LAG-3 specific signals from microglia. Antibodies can bind Fcγ receptors, so we blocked the suspected binding of antibodies to Fcγ receptors by preincubating microglia with CD16/CD32 antibody and/or mouse IgG [18]. However, none of these procedures would lower the fluorescence signals from isotype control antibody-treated microglia, thus the binding of antibodies to Fcγ receptors was not responsible for the fluorescence signals from FITC conjugated isotype control antibody-treated microglia (Fig. 2A). We then suspected that the autofluorescence may be responsible for the background signals. Indeed, there was no difference in fluorescence intensities between non-treated microglia and FTIC conjugated isotype control antibody-treated ones (Fig. 2B**)**. Therefore, autofluorescence likely causes the high fluorescent background in microglia and possibly lead to the failure of FITC conjugated LAG-3 antibody to detect LAG-3 protein in microglia.

We then tested LAG-3 antibodies conjugated with other fluorophores than FITC. Our results showed that PE-conjugated LAG-3 antibody significantly improved the sensitivity of detecting LAG-3 protein in microglia (Fig. 2C, D**)**. The ratio of median fluorescence intensity (MFI) of microglia treated with LAG-3 antibodies to its IgG control antibody, an indicator of sensitivity, was highest when antibody was conjugated with PE, while FITC and APC conjugated antibodies did not show a significant difference (Fig. 2E). Therefore, among commonly used fluorophores, PE-conjugated antibody outperforms APC and FITC conjugated antibodies in detecting surface molecules in microglia, by overcoming the interference from microglia autofluorescence.

**Fig. 3:**
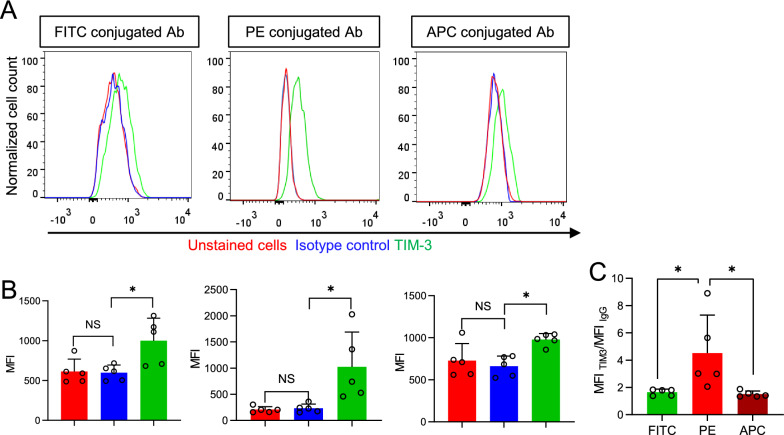
PE-conjugated antibody outperforms FITC and APC conjugated antibodies in detecting microglia TIM-3.** A** Distribution of fluorescence intensities from nontreated microglia, FITC, PE, and APC conjugated TIM-3 antibodies treated, and their respective isotype control antibody-treated microglia. **B** The MFI of microglia treated as in (**A**). **C** Comparison of MFI _TIM-3_/MFI _IgG_ of FITC, PE, and APC conjugated TIM-3 antibodies (n = 5 mice). Bars represent means ± SD. Comparisons were made by one-way ANOVA test with Tukey’s post hoc multiple comparisons test. *P < 0.05; **P < 0.01; NS, not significantly different

We extended our observation to the detection of T cell immunoglobulin-3 (TIM-3), another immune checkpoint receptor in microglia by flow cytometry. RNA-seq data and the study from Anderson et al. also suggest TIM-3 to be expressed in microglia [17, 19]. PE-conjugated TIM-3 antibody presented TIM-3 specific signals and yielded higher sensitivity for detection than FITC and APC conjugated antibodies (Fig. 3A, B). Therefore, regardless of the detection targets, PE-conjugated antibody yields higher sensitivity than FITC or APC conjugated antibody to detect protein expression in microglia.

### Microglia autofluorescence on fixed brain slices and its spectral properties

**Fig. 4:**
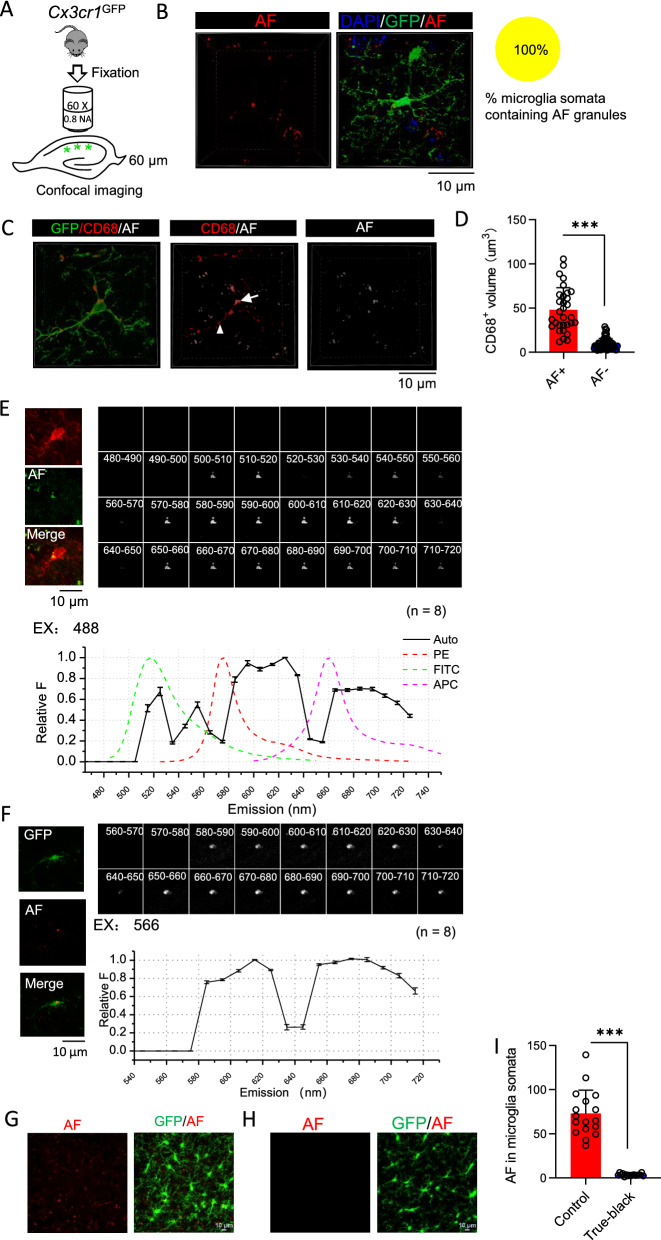
Imaging of autofluorescent granules in microglia on fixed brain slices. **A** Diagram illustrates the imaging of hippocampus CA1 microglia on fixed brain slices. **B** Representative 3D reconstructed images of a microglia from 2 months old *Cx3cr1*^GFP^ mice with autofluorescence (AF) imaged in TRITC channel (561 nm laser, 570–620 nm band pass filter). **C** Representative 3D reconstructed images of a microglia stained with CD68 from *Cx3cr1*^GFP^ mice. The arrow points to a CD68^+^ lysosome containing an autofluorescent granule in soma (AF +); while the arrowhead points to CD68^+^ lysosomes located in process without autofluorescent granules (AF-). **D** Volume of AF^+^ and AF^−^ CD68 + lysosomes as in panel (C) (n = 15 cells from 3 mice, Mann–Whitney test). **E** Images of an autofluorescent granule from a GFP expressing microglia through continuous emission filter excited by 566 nm laser and its corresponding emission spectrum (n = 8 cells from 3 mice). **F** Images of an autofluorescent granule from a microglia in wild type mouse labeled by Iba-1 antibody through continuous emission filter excited by 488 nm laser and its corresponding emission spectrum. The emission spectrum of FITC, PE, and APC were shown for comparison (n = 8 cells from 3 mice). **G** Z projected stack images of microglia from *Cx3cr1*^GFP^ mouse with autofluorescence imaged as in panel (B). **H** Z projected stack images of microglia on slices with the treatment of TrueBlack^®^ lipofuscin autofluorescence quencher before imaging. **I** Values of autofluorescence in microglial somata as in (**G**) and (**H**) (n = 17 cells from 3 mice, 2 tailed *t*-test). Bars represent means ± SD. ***P < 0.001

We expanded our study to identify the source of autofluorescence in microglia from 7–9 weeks old mice and studied its impacts on fluorescence imaging of microglia cells in fixed brain tissues. We imaged microglia in the stratum radiatum of the hippocampus CA1 region from fixed 7–9 weeks old *Cx3cr1*^GFP^ mice (Fig. 4A). We could observe autofluorescent granules inside the cytoplasm of GFP positive cells by imaging fluorescence signals excited by a 561 nm laser and emitted through a 570–620 nm band pass filter **(**Fig. 4B). Out of 16 microglia that we imaged from three mice, all contain autofluorescent granules (Fig. 4B). Autofluorescent granules are located in CD68 positive lysosomes in microglial somata or processes close to somata. There are also CD68 positive lysosomes which do not contain autofluorescent granules in both somata and processes (Fig. 4C). Lysosomes containing autofluorescent granules are significantly larger than those without autofluorescent granules (Fig. 4D).

We then applied spectral imaging on fixed brain slices to characterize the emission spectrum of microglia autofluorescence. Autofluorescence images were taken through a 400–720 nm continuous band pass filter, excited by a 488 nm laser or 561 nm laser. When excited by a 488 nm laser, autofluorescence from immunostained Iba-1 positive cells displayed multiple peaks on the emission spectrum (500–520 nm, 540–550 nm, 570–630 nm, 650–690 nm) (Fig. 4E). When excited by a 566 nm laser, autofluorescence from GFP expressing cells of *Cx3cr1*^GFP^ mice displayed an emission spectrum similar to that excited by 488 nm laser with peaks at 570–630 nm and 650–690 nm (Fig. 4F).

Due to their location and wide emission spectrum, these autofluorescent granules can be misidentified as fluorescent signals from immunofluorescence staining, thus we tested how to remove these autofluorescent signals. We found that they could be removed by TrueBlack^®^ lipofuscin autofluorescence quencher commercially available from Biotium (Fig. 4G–I).

### Autofluorescence can decrease calcium signals imaged by GCaMP6s on live brain slices

**Fig. 5:**
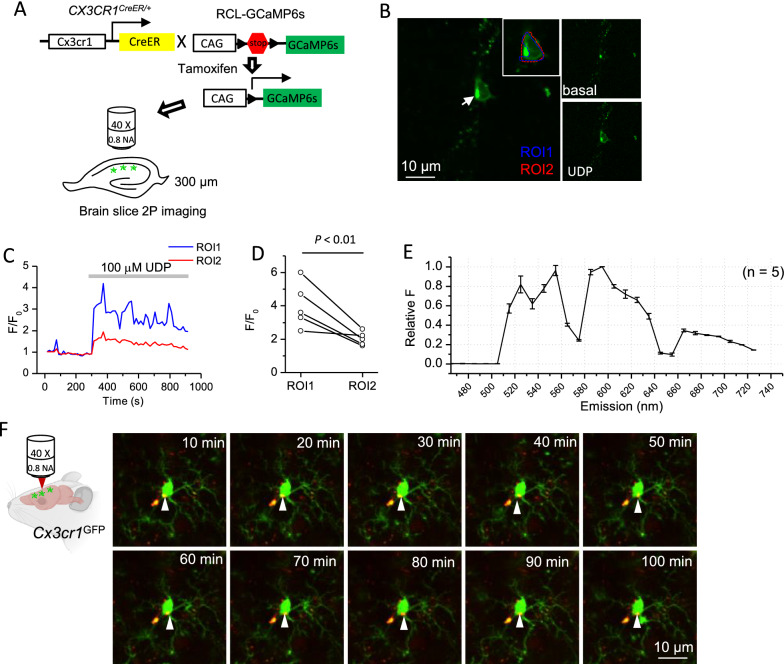
Imaging of autofluorescent granules in microglia on live slices and in vivo.** A** Diagram illustrates the strategy to image calcium signals in hippocampus CA1 microglia cells on live brain slices; B A representative Z-projected image of 60 GCaMP6s frames during 15 min imaging of a microglia cell on *Cx3cr1*^CreER^: GCaMP6s mouse brain slice. The arrow points to the soma of a microglia cell, which contains an autofluorescent granule. ROI1 circles the soma of microglia, excluding the autofluorescent granule, while ROI2 including it. Right top: the GCaMP6s image in basal condition. Right bottom: GCaMP6s image during application of 100 µM UDP. **C** F/F_0_ in ROI1 and ROI2 during 15 min imaging, at 5 min of which 100 µM UDP was applied. **D** The peak of F/F_0_ of GCaMP6s signals during UDP application in ROIs either excluding (ROI1) or including (ROI2) the autofluorescent granules (n = 5 cells from 3 mice, paired t-test). **E** Emission spectrum of the autofluorescent puncta as in panel (**B**) by spectral imaging (n = 5 cells from 3 mice). **F** Representative images of a microglia from *Cx3cr1*^GFP^ mice with GFP and autofluorescence simultaneously imaged by 2-photon microscopy through cranial window. The white arrows point to the autofluorescent granule in different time points. Bars represent means ± SD

At last, we characterized how autofluorescent granules would affect fluorescence imaging on ex vivo brain slices and in vivo. On live brain tissues, we imaged calcium signals in microglia from *Cx3Cr1*^Cre/ER^: GCaMP6s mice, which selectively express genetically encoded calcium indicator (GECI) GCaMP6s in microglia (Fig. 5A) [20, 21]. The application of 100 µM UDP can increase calcium signals in microglia, indicated by the increase of GCaMP6s fluorescence signals from microglia in the hippocampus CA1 region (Fig. 5B). Autofluorescent granules, which do not change their fluorescence intensities during UDP application, can be readily observed in GCaMP6s expressing microglia before UDP application (Fig. 5B). The amplitudes (F/F_0_) of calcium signals induced by UDP application were significantly lower if autofluorescent granules were included to quantify the GCaMP6s fluorescent signal in microglial somata (Fig. 5C, D). Therefore, autofluorescent granules need to be excluded when selecting regions of interests (ROI) to quantify the amplitudes of somatic calcium signals in microglia. We also took the opportunity to acquire the emission spectrum of autofluorescent granules from GCaMP6s expressing microglia on live brain slices, revealing emission spectrum with patterns similar to those acquired on fixed slices (Fig. 5E).

We also imaged these autofluroescent granules in vivo from anesthetized 9–11 weeks old *Cx3cr1*^GFP^ mice by single wavelength 2-photon microscopy. We optimized the strategy to image autofluorescent granules by single-wavelength 2-photon microscopy and determined that exciting both at 820 nm and collecting GFP signals through 500–550 nm filter, while autofluorescent signals through 600–656 nm filter produced the images with the most balanced quality between GFP and autofluoresence. We tracked the location of autofluorescent granules in microglia for 90 min with 10 min interval and observed that they displayed restricted movement inside microglial somata (Fig. 5F; Additional file 2: video S1). This experiment also proved it feasible to acquire long term images of autofluorescent granules in microglia to study their accumulation during aging.

## Discussion

In the report, we studied how autofluorescence would affect the application of fluorescence-based techniques to study microglia. First, we studied how flow cytometry would be affected by microglia autofluorescence. We systematically compared the sensitivity of FITC, PE and APC conjugated antibodies to detect protein expression in microglia by flow cytometry and found that PE-conjugated antibody has the highest sensitivity to detect protein expression in microglia. Therefore, due to the strong autofluorescence from microglia, the fluorophores conjugated to the antibody for flow cytometry need to be carefully selected to study microglia. The optical properties of the microglia autofluorescence and the antibodies conjugated fluorophores could both account for the higher sensitivity of PE-conjugated antibodies to detect medium or low abundant protein expression in microglia. PE has a relatively higher quantum yield than FITC and APC and thus was more capable of overcoming interference from autofluorescence. Our spectral imaging on microglia autofluorescence revealed an emission spectrum with multiple peaks, consistent with the fact that lipofuscin is a mixture. According to the emission spectrum of autofluorescence excited by 488 nm laser, the emission peak of PE (~ 570 nm) overlaps with one of the troughs of microglia autofluorescence emission spectrum, offering another explanation why PE outperforms other fluorophores (Fig. 4E).

We also found that the isotype control antibody (rat IgG1, kappa), which we have tested, doesn’t bind to microglia, thus eliminating the necessity to use blockers, such as the CD16/CD32 antibody, to block the potential binding of antibodies to Fcγ receptors. The CD16/CD32 antibody can block FcγRs I, IIb, and III, while CD16.2 antibody can bind FcγRIV. However, in our case, rat IgG1 may not bind to either type of these Fcγ receptors. But it needs to be mentioned that this may not apply to antibodies with isotypes other than rat IgG1, especially because Biburger et al. reported that antibodies of different isotypes can have varying binding capacities to FcγRIV [18].

Lipofuscin-like autofluorescent granules have been reported to be accumulated in microglia in aged animals [11, 14]. Here we reported that autofluorescent granules can be observed in both fixed and live brain slices from young mice. Our experiments strongly supported that the substance emitting autofluorescence is lipofuscin. First, microglia autofluorescence is localized in cytoplasmic granules, consistent with lipofuscin stored in lysosomes; second, microglia autofluorescence can be removed by lipofuscin autofluorescence quencher; third, the multi-peak emission spectra of microglia autofluorescence agrees with the fact that lipofuscin is a mixture of partially digested proteins and lipids. To our knowledge, it is still unknown how lipofuscin was produced. Safaiyan et al. have suggested microglia lipofuscin was derived from ingested myelin fragments, but the study has not provided enough evidences to support this notion [13]. Another study suggested that lipofuscin was derived from mitochondria and the proteome study also supported that lipofuscin contains mitochondria protein [22, 23]. Since ageing process should not affect the mechanism of lipofuscin production, we suspect that the optical properties of lipofuscin should not change dramatically during the aging process.

Autofluorescent granules in microglia from fixed brain slices of young mice, where immunofluorescence staining is usually performed, warn us to be cautious when staining the intracellular antigens in microglia by immunofluorescence. It could be a concern that the autofluorescent puncta may be misidentified as engulfed materials, had proper control experiments not been performed. It has been suggested that microglia can engulf molecules, such as PSD95, synaptophysin, and C1q, commonly by immunofluorescence showing their co-localization with CD68^+^ lysosome structure [24–26]. Our study has shown that autofluorescence quencher, such as the one from Biotium we used in this study, can remove the microglia autofluorescence, and thus should help the identification of intracellular puncta staining in microglia. The alternative strategy is to use antibody conjugating fluorophores with emission peaks overlapping with the troughs of autofluorescence emission spectrum. According to our acquired emission spectrum of microglia autofluorescence, fluorophores such as Alexa555 (peak at 568 nm) or Alexa 633 (peak at 650 nm) should be able to overwhelm autofluorescence, when combined with an appropriate emission filter.

Live imaging of calcium signals in microglia has been performed by either loading calcium dye into the cells or selectively expressing GECIs in them [7–9]. The reason why autofluorescent granules have not been observed or reported in these studies can be the strong fluorescence emitted from calcium indicators or other fluorophores in these studies. The concentration of organic dye loaded into microglia cells by electrophoresis can be high enough to mask the endogenous autofluorescent granules. We imaged calcium signals in microglia by selectively expressing GCaMP6s in microglia. Due to the low basal calcium level, autofluorescent granules can be readily observed in microglia before UDP application. Umpierre et al. used a similar strategy to express GCaMP6s in microglia, but with another *Cx3Cr1*^CreER^ mouse line, which expresses YFP in microglia and therefore may mask the autofluorescent granules [9].

Our study showed that autofluorescent granules exist in microglia from young mice, and they have profound impacts on fluorescence-based methods, including flow cytometry, immunofluorescence, and live imaging. Microglia autofluorescence can affect the sensitivity of flow cytometry to detect protein expression. Among PE, FITC and APC, PE-conjugated antibody has the best ability to overcome the interference from microglia autofluorescence. The existence of autofluorescent granules in microglia also requires us to design immunofluorescence experiments and interpret their results with care. Our live imaging experiments confirmed the existence of autofluorescent granules in microglia and suggested that they can decrease the sensitivity of detecting Ca^2+^ signals in microglial somata.

## Supplementary Information

**Additional file 1: Fig. S1** RNA-sequencing data suggests LAG-3 and TIM-3 expression in microglia. Fragments per kilobase million (FPKM) of LAG-3 (**A**) and TIM-3 (**B**) expression in mouse brain cells was extracted from brainrnaseq.org, an online data repository for an RNA-sequencing transcriptome and splicing database of cells in the cerebral cortex [17].

**Additional file 2: Video S1. **In vivo imaging of somatosensory cortex microglia. A representative video of 90 min in vivo imaged microglia from *Cx3cr1*^*GFP*^ mice. GFP was shown in green, and autofluorescence was shown in red.

## Data Availability

The datasets used and/or analyzed during the current study are available from the corresponding author on reasonable request.

## Abbreviations

FITC: Fluorescein isothiocyanate
PE: Phycoerythrin
APC: Allophycocyanin
LAG-3: Lymphocyte-activation gene 3
TIM-3: T cell immunoglobulin-3
ROI: Region of interests
