# Supplementary figures and images for: Impacts of autofluorescence on fluorescence based techniques to study microglia
Source: BMC Neurosci. 2022 Mar 31;23:21. doi: 10.1186/s12868-022-00703-1 (PMC8973892; doi:10.1186/s12868-022-00703-1)

A

B


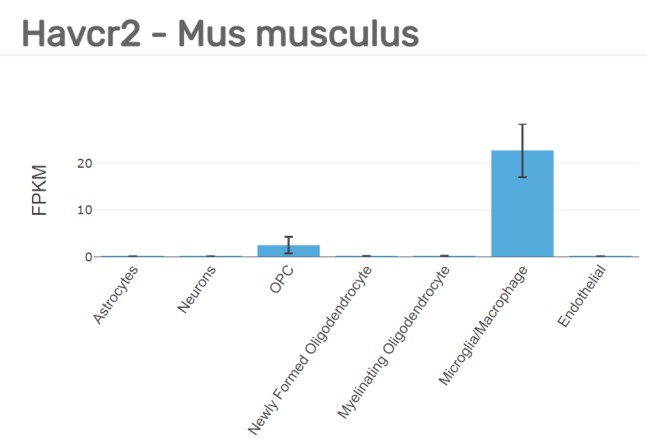

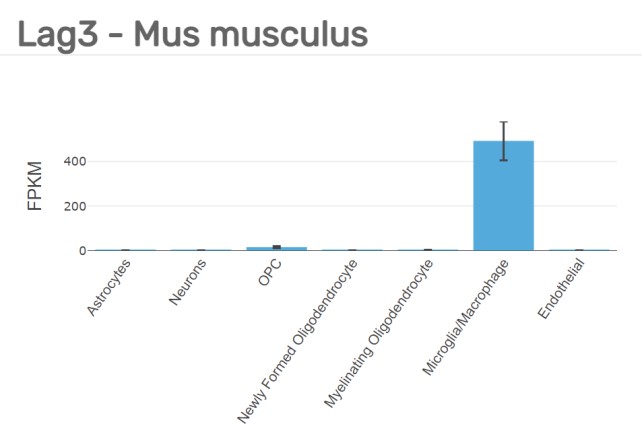


Zhang & Xu, supplementary Figure 1

Supplement: Supplementary file 1 — Additional file 1: Fig. S1 RNA-sequencing data suggests LAG-3 and TIM-3 expression in microglia. Fragments per kilobase million (FPKM) of LAG-3 (A) and TIM-3 (B) expression in mouse brain cells was extracted from brainrnaseq.org, an online data repository for an RNA-sequencing transcriptome and splicing database of cells in the cerebral cortex [17]. [file 12868_2022_703_MOESM1_ESM.docx]
